# The Texas Medical News

**Published:** 1896-06

**Authors:** 


THE *Texas Medical News* for May (late *Texas Sanitarian*), J. W. McLaughlin and T. J. Bennett editors (McLaughlin *loquitur*), says:

"Is it not strange that Dr. Daniel, in the May number of the TEXAS MEDICAL JOURNAL, should say that 'the legislature has no authority to transfer the cost of quarantine to the United States government,' when he, as secretary of the State Health Officer, must have known that the cost of quarantine at Camp Jenner was so transferred by the Health Officer," etc. * * * "Dr. Daniel, as Secretary of this officer, must have known this, and his remarks * * * should be taken *cum grano salis.*"

Not at all strange; because it is *true.* The legislature has *no authority to transfer the cost of State quarantine* to anybody; nor did the State Health Officer "so transfer the cost of quarantine at Camp Jenner." The quarantine at Eagle Pass (near which station Camp Jenner was established) was kept in full operation during and after the refugee episode, the State Quarantine Officer, Dr. Evans, in charge, on full pay, inspecting every train, and no one being admitted to nor discharged from quarantine except on his order, all the expense of quarantine being paid by the State, except the expense of caring for the refugees, which, *alone*, was turned over to the Marine Hospital Service.

The camp was established by State Health Officer Swearingen, and this army of refugees was being fed and cared for by the State. The United States government wanted them better fed and better cared for, and offered assistance, and was told to go ahead. Dr. Magruder, of the Marine Hospital Service, named the camp "Camp Jenner," and the government furnished nurses, tents and supplies, which was right and proper under the circumstances, the aim of the law being to aid and co-operate with State quarantine. But had the Marine Hospital Service not done so, the State could, and would have cared for these people, even though it had been necessary to go in debt, and there is not a question but that the debt would have been cheerfully paid by special appropriation by the next legislature.

The *Texas Medical News* might just as well, and with equal propriety, have cited as an instance of "transferring the cost of State quarantine to the United States government," the case of an infected ship arriving at some minor station. At such station there are not proper facilities for quarantining the passengers, shifting cargo and disinfecting the vessel, cargo, passengers and crew, and in every such instance the vessel is, *in accordance with the law made and provided for such cases*, ordered to some Federal quarantine station for treatment and care, the expense falling on the Marine Hospital Service. That is done every summer in nearly every coast State. In no instance, except at Brunswick, as stated by us, has a *State quarantine* been taken charge of by the United States authorities, and the State officers relieved, or the State relieved of the expense of quarantine. The case of Camp Jenner is in no sense a parallel case, and furnishes neither precedent nor illustration of "transferring the cost of State quarantine to the United States government."

Were it worth while to say more on the subject on which the doctor has gone astray, it can be shown that there is nothing in any law, anywhere, State or Federal, that provides for a "transfer of State quarantine expense to the general government." The only possible way in which it could be done would be by petition to congress for an act for the relief of the poor old

State of Texas. For our part, we could never submit to the humiliation of being the first, nor, as to that matter, the last State to plead the insolvent act; it would be a false pretence; it is not a matter of ability at all. Poor little Rhode Island would not ask such a thing.

The only thing in either State of Federal law bearing upon this subject at all,—and it certainly does not provide for any transfer of the *cost of maintaining* State quarantine to the general government,—is found in Section 8 of the Quarantine Laws and Regulations of the United States, approved February 15, 1893. It reads at follows:

"Whenever the proper authorities of a State shall surrender to the United States *the use* [italics ours.—Ed.] *of* THE BUILDINGS and disinfecting apparatus of a State quarantine station, the Secretary of the Treasury shall be authorized to receive them, and to *pay a reasonable compensation to the State for their* USE [italics and small caps. ours.—Ed.], if, in HIS OPINION, THEY ARE NECESSARY to the United States."

And this is cited by Dr. McLaughlin, editor, in the February, '95, issue of the *Texas Sanitarian*, as "showing [page 166] that the surrender of a State's maritime quarantine is comtemplated by the Federal authorities"!!! And this, too, even after the Surgeon General M. H. S., in reply to an inquiry, had written the *Texas Sanitarian* that "THERE IS NO APPROPRIATION under the control of the Marine Hospital Service *for paying the current expenses of* ANY quarantine station in addition to those already in operation. ADDITIONAL APPROPRIATIONS will have to be made [by special act of congress, mind you.—Ed.] to provide for any new stations."

It looks as if this should have settled the matter, and silenced a less zealous and disinterested party. It was disingenuous in Dr. McLaughlin not to explain that Section 8 referred to an antecedent section (part of Section 3), which provides that "if in the opinion of the Secretary Treasury" a State quarantine is not sufficiently rigid, the Secretary of the Treasury "may make *additional rules and requirements*,—may strengthen the State quarantine,—and "if the State or municipal authorities shall fail or refuse to enforce SUCH RULES AND REGULATIONS, the President shall execute and enforce them, * * * and may detail or appoint officers for that purpose" (that is, of enforcing the *additional rules:* even this does not supersede the State's authority, nor relieve the State of expense). Here then, is where the provisions of Section 8 come in—for paying the State *rent* for the buildings and apparatus, "if in the opinion," etc., the *use* of the same "is necessary to the United States."

See how many contingencies are here attached. And yet Section 8 is quoted to show that if the Texas Legislature will "Transfer" the expense of maintaining State quarantine, the United States government is ready and willing to assume said expense. We cannot see upon what this assumption is predicated. Is that not pretty far fetched? the small blanket considerably stretched? It reminds us of the nursery story of the little chicken who gave the alarm that the sky was falling because a rose leaf had fallen on his tail.

